# Fast hybrid methods for modeling landslide susceptibility in Ardal County

**DOI:** 10.1038/s41598-024-53120-1

**Published:** 2024-02-06

**Authors:** Shangshang Xu

**Affiliations:** School of Geography and Environmental Science, University of Southampton, Southampton, SO17 1BJ UK

**Keywords:** Environmental sciences, Environmental social sciences, Solid Earth sciences

## Abstract

Recently, machine learning models have received huge attention for environmental risk modeling. One of these applications is landslide susceptibility mapping which is a necessary primary step for dealing with the landslide risk in prone areas. In this study, a conventional machine learning model called multi-layer perceptron (MLP) neural network is built upon advanced optimization algorithms to achieve a firm prediction of landslide susceptibility in Ardal County, West of Iran. The used geospatial dataset consists of fourteen conditioning factors and 170 landslide events. The used optimizers are electromagnetic field optimization (EFO), symbiotic organisms search (SOS), shuffled complex evolution (SCE), and electrostatic discharge algorithm (ESDA) that contribute to tuning MLP’s internal parameters. The competency of the models is evaluated using several statistical methods to provide a comparison among them. It was discovered that the EFO-MLP and SCE-MLP enjoy much quicker training than SOS-MLP and ESDA-MLP. Further, relying on both accuracy and time criteria, the EFO-MLP was found to be the most efficient model (time = 1161 s, AUC = 0.879, MSE = 0.153, and *R* = 0.657). Hence, the landslide susceptibility map of this model is recommended to be used by authorities to provide real-world protective measures within Ardal County. For helping this, a random forest-based model showed that Elevation, Lithology, and Land Use are the most important factors within the studied area. Lastly, the solution discovered in this study is converted into an equation for convenient landslide susceptibility prediction.

## Introduction

With the advent of advanced computational and simulation techniques, many fields of research have been revolutionized toward attaingng more comprehensive solutions^1–4^. Environmental risk assessment is one of these fields that has experienced considerable improvement in recent decades^5^. Landslide susceptibility assessment is one of these fields that plays an imperative role in harnessing this hazard in prone areas^6,7^. As a prerequisite of many landslide susceptibility mapping approaches, a spatial database must be provided that contains the records of historical landslides, as well as relevant landslide conditioning factors (LCFs)^8–10^. Analyzing the spatial relationship between the landslide occurrence and different LCFs is the basis of studies^11^ which provide a predictive landslide susceptibility map for a specific area within environments such as geographic information system (GIS) and Google earth engine (GEE)^12,13^.

As far as the approach is concerned, the spatial relationship between the LCFs and historical landslides can be investigated based on various methods including statistical analysis^14^, deep learning^15^, decision-making^16^, etc. For instance, statistical index (SI)^17^, weights of evidence (WoE)^18^, and frequency ratio (FR)^19^ are among the most popular statistical models for this purpose. As for decision-based models, analytical hierarchy process (AHP)^20^ is a popular approach that draws on relative experts’ ratings to the LCFs.

Machine learning models, however, have been recently famous due to their several advantages^21–24^. In general, these models are capable to non-linearly analyze the dependency of one (or more) target parameter(s) on several influencial factors and create a predictive model in various domains^25–27^. As for landslide hazard assessment, machine learning models can explore how historical landslides occurred under the effect of LCFs, and accordingly, predict landslide susceptibility values (V_LS_) to generate the maps. Artificial neural network^28^, random forest^29^, regression models^30^, support vector models^31^, and fuzzy-based models^32^ are prevalent machine learning methods that have been used for landslide susceptibility modeling in different parts of the globe. However, many scholars have suggested to enhance the efficiency of these models by incorporating optimization algorithms, a.k.a metaheuristic algorithms^33,34^. Popular optimization algorithms such as particle swarm optimization (PSO)^35^, genetic algorithm (GA)^36^, and differential evolution (DE)^37^ have been extensively used for optimizing the computational parameters of machine learning models which are responsible for tuning the LCFs-landslide relationships. Newer generations of optimization algorithms consist of Harris hawks optimization (HHO)^38^, salp swarm algorithm (SSA)^39^, cuckoo optimization algorithm (COA)^40^, Satin bowerbird optimizer (SBO)^41^, teaching–learning-based optimization (TLBO)^42^, biogeography-based optimization (BBO)^43^, etc. which have served for landslide susceptibility mapping worldwide. These models provide an optimum response for the training of machine learning models, and hereby, the prediction is protected against drawbacks such as local minima. However, being time-consuming has been frequently mentioned as a critical disadvantage of the models built upon optimization algorithms. Therefore, it calls for trying more time-effective methods for high dimensional problems such as landslide susceptibility mapping.

Iran is one of the most landslide-susceptible countries in the world^44^. The northern and western parts of Iran are considered mountainous areas due to the presence of the Alborz and Zagros mountains. For this reason, many relevant studies have focused on study areas located in northern and western Iran^45–47^. Speaking of western parts, Chaharmahal va Bakhtiari Province is among the most hazardous areas with many historical landslides that affect human life and assets^48,49^. Therefore, employing time-efficient methods to develop accurate susceptibility maps within this province is a necessary task.

This study aims to provide a fast yet accurate hybrid machine learning algorithm for landslide susceptibility mapping at Ardal County, Chaharmahal va Bakhtiari Province in Iran. To create this model, an MLP neural network is trained using an optimization technique called electromagnetic field optimization (EFO)^50^ to develop the EFO-MLP ensemble. The advantage of the proposed model compared to the previous hybrid machine learning algorithms lies in its high convergence speed and powerful optimization. A comparative manner is adopted to validate the competency of the EFO-MLP model. In so doing, the MLP is likewise ensembled with another quick algorithm, namely shuffled complex evolution (SCE)^51^, as well as two others, namely symbiotic organisms search (SOS)^52^ and electrostatic discharge algorithm (ESDA)^53^ which possess high optimization ability. The suggested model will be compared to similar ones from the previous studies and a certified mathamtical formula is extracted from it for practical usages. The resulting landslide susceptibility maps can be used by relevant policy-makers in Ardal County to better prepare for future landslides. Another pivotal outcome of this work will be analyzing the sensitivity of landslide occurrence to a wide range of LCFs and determining the most important ones.

## Data

### Study area and landslide inventory

**Figure 1:**
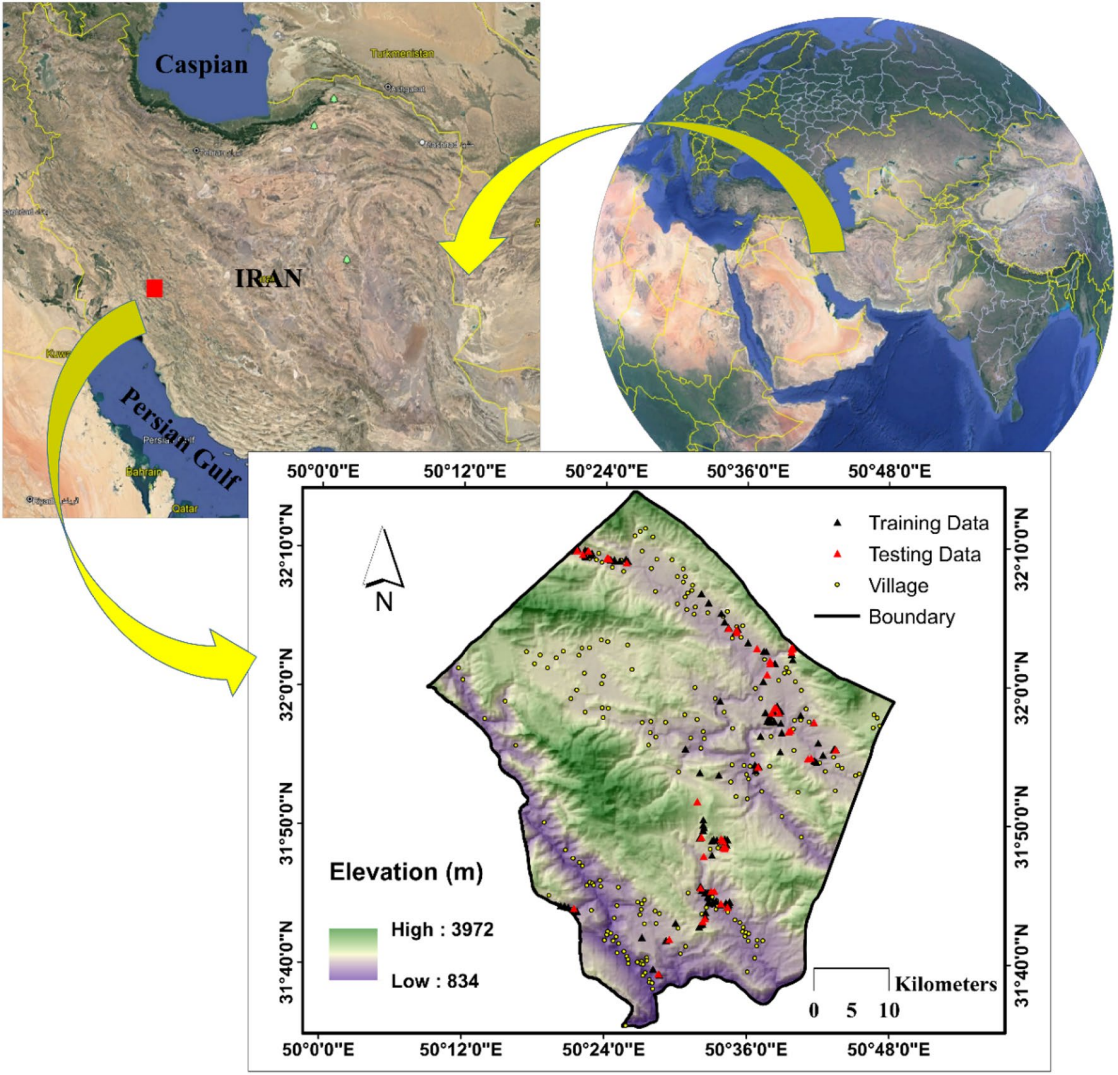
Location of Ardal County and the distribution of the villages and historical landslides—created using ArcGIS v10.5^54^ and Google Earth Pro v7.3.1^55^.

Ardal County is a part of Chaharmahal-va-Bakhtiari Province located in the western part of Iran (Fig. 1). As is known, this part of Iran is characterized by the presence of the Zagros Mountains making it a cold and high-altitude area. The mean annual temperature is about 15 °C and owing to the abundant precipitations in winter, the climate of this area is mostly Humid.

As per Fig. 1, the study area falls within the longitude from 50° 09' to 50° 48' E and the latitude from 31° 35' to 32° 14' N. Forests and ranges cover around 67% of this area. Based on the geological map, around 37% of the area is settled on Undivided Bangestan Rock, mostly including limestone and shale. Moreover, the soil type map reports that the majority of the soil in this area is labeled as Rock Outcrops/Entisols.

The landslide inventory map was obtained from the archive of the national geoscience database of Iran (NGDIR). After clipping for this specific area, 170 landslides were contained which have mostly occurred along the roads and rivers, and near the human residences (i.e., villages). Hence, the landslides in this area are a potential danger to both human lives and infrastructures. The dominant types of these slides were translational and lateral spread landslides. Figure 1 shows the location of landslides with two categories: (i) black triangles which represent the landslide events considered for training the models and (ii) red triangles which represent the landslide events for testing the prediction competency of the trained models. These points were randomly selected with a 70/30 ratio specifying 119 and 51 landslides in the training and testing phases, respectively.

### Landslide conditioning factors and importance analysis

Fourteen LCFs are considered in this research to affect the susceptibility of landslide. These factors include Elevation, Slope Aspect, Slope Degree, Profile Curvature, Plan Curvature, Distance to three linear phenomena (i.e., D. to Faults, Roads, and Rivers), Soil Type, Lithology, Land Use, Topographic Wetness Index (TWI), Stream Power Index (SPI), and Normalized Difference Vegetation Index (NDVI). Apart from some local maps, the main resources of these LCFs were the digital elevation model (DEM) of the study area and the maps provided by the Iranian Soil Conservation and Watershed Management Research Institute (SCWMRI) and Geological Society of Iran (GSI). All data was processed and analyzed within GIS to create the relevant layers. Table 1 presents the classes/categories of the LCFs along with the corresponding source. Note that the classification of the layers has been performed with reference to previous studies^49^.

**Table 1:** Classification details of the used GIS layers.

LCF	Classes/categories	Source	LCF	Classes/categories	Source
Elevation	< 1000	DEM	D. to Rivers	(0–150)	Local maps
(m)	(1000–1500)		(m)	(150–300)	
	(1500–2000)			(300–450)	
	(2000–2500)			(450–600)	
	(2500–3000)			> 600	
	> 3000				
Slope Aspect	North	DEM	Soil type	Rock Outcrops/Entisols	SCWMRI
	North-East			Inceptisols	
	East			Rock Outcrops/Inceptisols	
	South-East				
	South				
	South-West				
	West				
	North-West				
Slope Degree	< 5	DEM	Lithology	L1–L16 (see S1)	GSI
(°)	(5–10)				
	(10–20)				
	(20–30)				
	(30–45)				
	> 45				
Profile curvature	Concave	DEM	Land Use	LU1–LU18 (see S2)	SCWMRI
	Flat				
	Convex				
Plan curvature	Concave	DEM	TWI	< − 1.8	(see ES1)
	Flat			(− 1.8–2.7)	
	Convex			(2.7–4.5)	
				(4.5–7.1)	
				> 7.1	
D. to Faults	(0–150)	GSI	SPI	< 1540.5	(see ES2)
(m)	(150–300)			(1540.5–6932.6)	
	(300–450)			(6932.6–17,331.7)	
	(450–600)			(17,331.7–37,359.5)	
	> 600			> 37,359.5	
D. to Roads	(0–100)	Local maps	NDVI	(− 0.14– − 0.02)	MODIS imagery
(m)	(100–200)			(− 0.02–0.03)	
	(200–300)			(0.03–0.05)	
	(300–400)			(0.05–0.07)	
	> 400			(0.07–0.27)	

**Figure 2:**
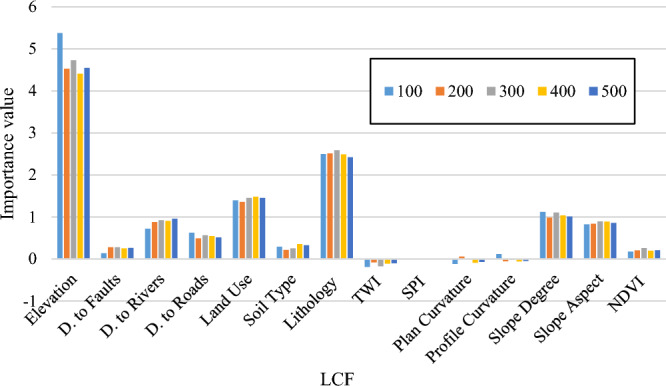
The results of RFIA for different tree ensembles.

The importance of each LCF for landslide susceptibility analysis in the selected area is investigated using a machine learning-statistical method. Random forest importance assessment (RFIA) is a well-known method that has served for this purpose in many studies^56^. In this model, a random forest^57^ is applied to the data and it produces importance values based on the sensitivity of the landslide occurrence to each LCF. The size of the forest is an important paremetr which tried to be 100, 200, 300, 400, and 500 for ensuring the repeatability of the results. Figure 2 shows the obtained values in the form of barcharts. As is seen, all tested populations agree that Elevation is the most important LCF in this study, followed by Lithology and Land Use. On the contrary, TWI, SPI, Profile Curvature and Plan Curvature received the lowest importance values.

## Methodology

### MLP neural network

The MLP is known as the most common and formidable type of conventional ANNs^58,59^. This model relies on a layered skeleton and connective weights between these layers. The first layer of each MLP is called the input layer. By receiving a numerical dataset with distinguished inputs and targets, the MLP sends each input parameter to one input node. These nodes then communicate with subsequent nodes lying in the middle layer(s) called the hidden layer. At the end, the model produces an estimation of the target parameter (called output) through the output layer.

The main calculations in an MLP are carried out by the hidden and output nodes. These nodes aim to explore the relationship between the inputs and target to learn the mathematical pattern behind. This process is accomplished by tuning the weights and biases of the MLP^60^. In each epoch, the network evaluates its accuracy by comparing the produced outputs with the target values. An error is calculated and the network propagates backward to adjust the weights and biases to minimize the error of prediction. That’s the reason these models are called backpropagation MLPs^61^.

**Figure 3:**
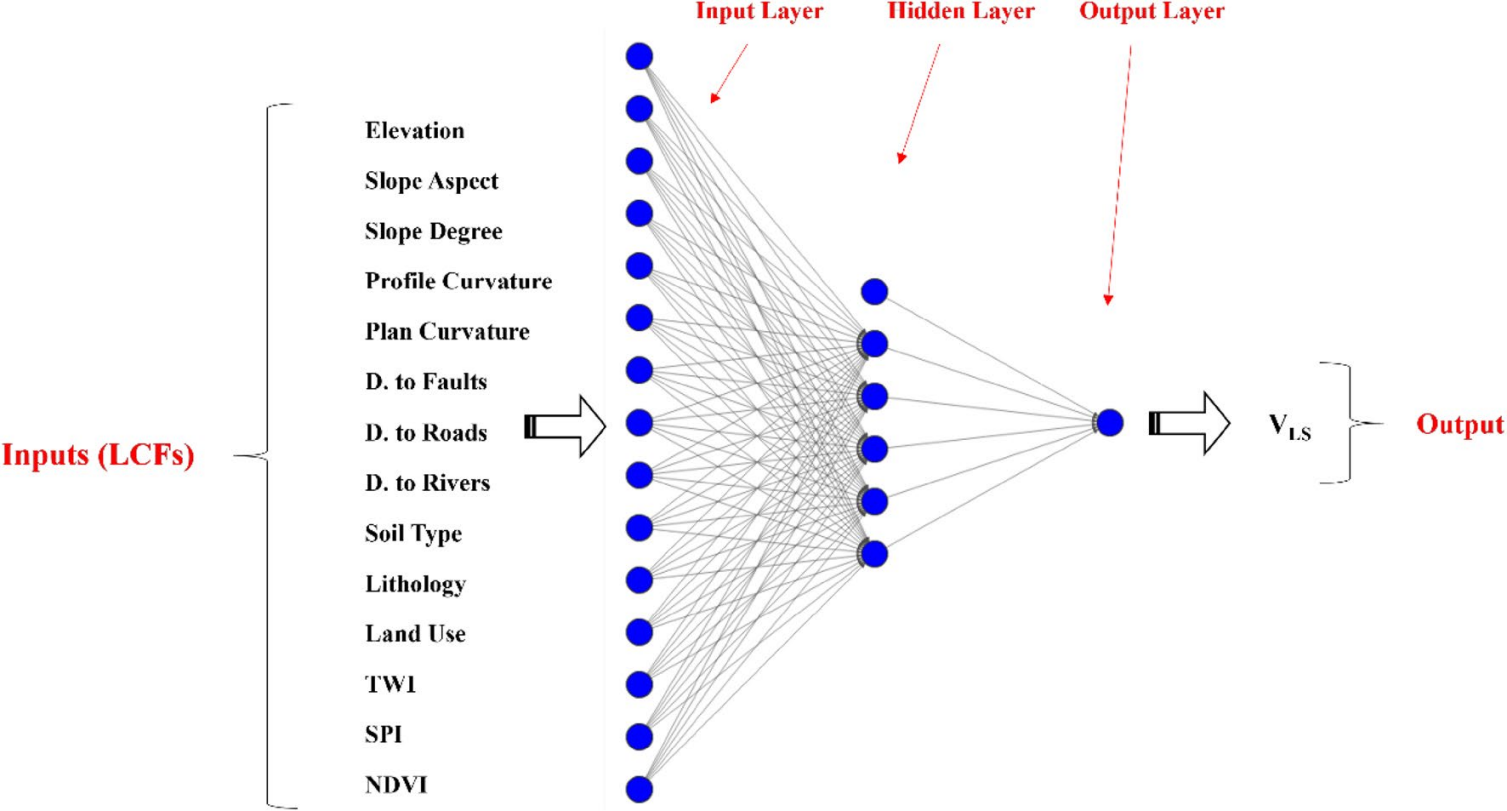
The configuration of the assigned MLP network.

Figure 3 shows the MLP model tailored for this study. In order to simplify the calculations, a three-layered network has been assigned to the task with 14 input nodes (one for each LCF), 5 hidden nodes, and 1 output node (for the V_LS_). Also, there are 5 bias terms for the hidden neurons and 1 bias term for the output neuron. The used activation functions for the hidden and output neurons are Tansig and Prelin, respectively, which are selected based on similar applications supported by trial-and-error efforts compared to other exising functions.

### Optimization algorithms

Four optimization algorithms have been selected to train the MLP model in predicting landslide susceptibility. These algorithms are population-based optimizers that seek the optimum solution to a problem in hand using a nature- or physics-based mechanism. The general explanation of the used optimizers is as follows:

1. The EFO, proposed by Abedinpourshotorban et al.^50^, is a nature-inspired optimization algorithm that draws its principles from the behavior of charged particles in an electromagnetic field. In EFO, potential solutions to an optimization problem are treated as charged particles, and their positions in the search space represent their states. The algorithm mimics the electromagnetic interactions among these particles, simulating the attraction and repulsion forces experienced by charged objects. Particle positions are updated iteratively based on these forces, with better solutions being attracted to others while avoiding overcrowding, similar to how charged particles move in response to electromagnetic forces.
2. The SCE algorithm was designed by Duan et al.^51^ to tackle complex, multidimensional search spaces. It operates by maintaining and evolving multiple solution complexes simultaneously. Each complex represents a collection of potential solutions. The SCE algorithm employs a mix of systematic and random perturbations to explore the solution space comprehensively. Systematic perturbations involve shifting and reshuffling solutions within and between complexes, emulating a natural selection process where better solutions tend to replace inferior ones. Random perturbations introduce variability, preventing the algorithm from getting stuck in local optima. SCE effectively balances exploration and exploitation of the search space, and its mechanism aims to continuously improve the quality of solutions over iterations.
3. The SOS was developed by Cheng and Prayogo^52^ as an optimization algorithm inspired by the mutualistic relationships observed in ecological systems. It emulates the interactions between different organisms, specifically mutualism and commensalism, to solve complex optimization problems. In SOS, potential solutions, represented as organisms, coexist in a population. Mutualistic pairs collaborate to enhance their fitness, while commensal organisms benefit without directly contributing. The algorithm evolves over iterations through a process of mutual benefit and random changes, wherein solutions sharing resources tend to improve together. This mechanism balances exploration (random changes) with exploitation (mutualistic improvements) and promotes the discovery of high-quality solutions in diverse problem domains. SOS showcases the power of mimicking natural ecological interactions to optimize complex systems efficiently.
4. The ESDA, designed by Bouchekara^53^, is inspired by the physics of electrostatic discharge. ESDA simulates the process of electrostatic discharge, where the accumulated electrical potential is suddenly released. In this algorithm, potential solutions to an optimization problem are analogous to charged particles. Like particles seeking a path to discharge their accumulated charge, solutions navigate towards better fitness values. ESDA leverages the principles of attraction and repulsion observed in electrostatic phenomena. Solutions with similar charges (fitness values) repel each other, encouraging exploration, while those with opposite charges are attracted, promoting exploitation. Over successive iterations, this mechanism leads to the convergence of solutions toward optimal or near-optimal solutions.

Detailed mathematical rules of the used algorithms can be sufficiently found in earlier literature, i.e.^62,63^, for the EFO^64,65^, for the SCE^66,67^, for the SOS, and^68,69^ for the ESDA.

### Accuracy criteria

A popular accuracy assessment method for the prediction of natural disasters is drawing the receiving operating characteristics curve and calculating the area under the curve (AUC)^70,71^. This area may range from 0.5 to 1.0 representing a random and ideal prediction, respectively. The calculation of the AUC value is associated with two other parameters, namely specificity and sensitivity which respectively reflect the ratio of the correctly classified non-landslide and landslide pixels. Therefore, the higher the AUC, specificity, and sensitivity, the more accurate the prediction. Below equations formulate these three indices.

1 $$Sensitivity = \frac{PT}{{PT + NF}}$$

2 $$Specificity = \frac{NT}{{NT + PF}}$$

3 $$AUC = \frac{\sum PT + \sum NT}{{LS + nonLS}}$$

where PT, PF, NT, and NF represent true positive, false positive, true negative, and false negative, respectively. Moreover, LS and nonLS stand for the total number of landslides and non-landslides, respectively.

In addition to these three indices, an error indicator called mean square error (MSE) and a correlation criterion called Pearson correlation coefficient (R) have been employed as per Eqs. 4 and 5.

4 $$MSE = \frac{1}{K}\mathop \sum \limits_{i = 1}^{K} \left( {X_{{i_{observed} }} - X_{{i_{predicted} }} } \right)^{2}$$

5 $$R = \frac{{\sum\limits_{i = 1}^{K} {\left( {X_{{i_{predicted} }} - \overline{X}_{predicted} } \right)\left( {X_{{i_{observed} }} - \overline{X}_{observed} } \right)} }}{{\sqrt {\sum\limits_{i = 1}^{K} {\left( {X_{{i_{predicted} }} - \overline{X}_{predicted} } \right)^{2} } } \sqrt {\sum\limits_{i = 1}^{K} {\left( {X_{{i_{observed} }} - \overline{X}_{observed} } \right)^{2} } } }}$$

where *X*_*i observed*_ and *X*_*i predicted*_ stand for the real and predicted V_LS_, respectively. Also, the number of evaluated pairs is represented by *K* in this equation.

### Hybrid modeling

The models that are proposed in this study are intelligent hybrids composed of an MLP neural network and an optimization algorithm (either EFO, SCE, SOS, or ESDA). Hence, the models are named EFO-MLP, SCE-MLP, SOS-MLP, and ESDA-MLP.

**Figure 4:**
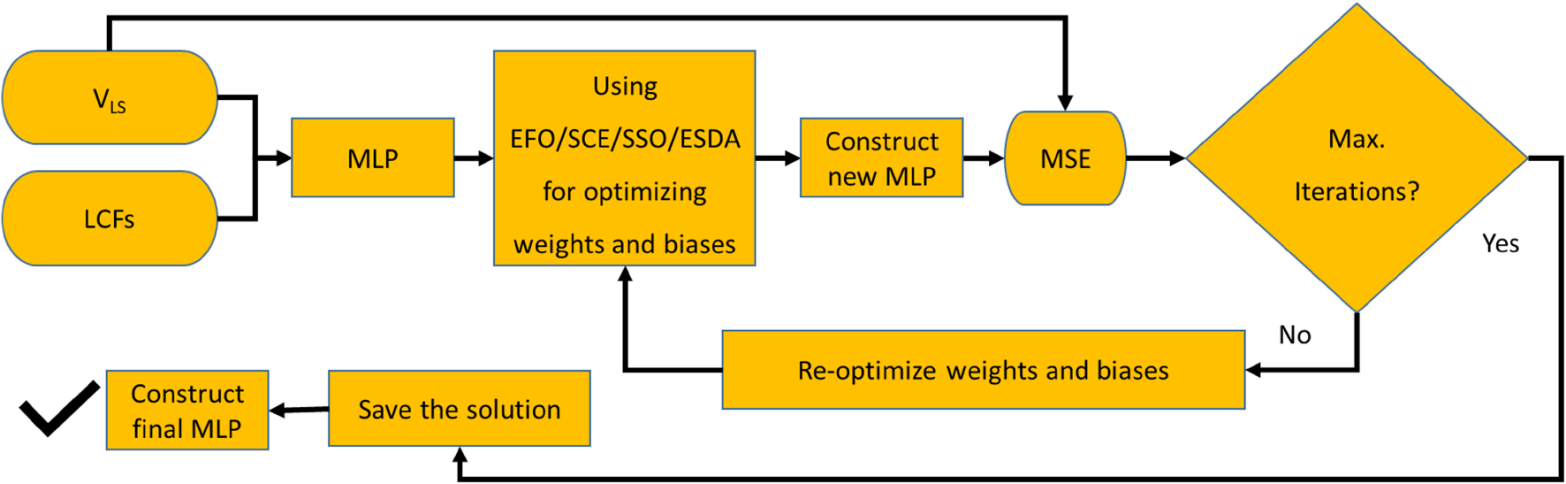
Optimization flowchart of the MLP.

As per Fig. 3, a regular MLP is responsible for receiving the LCFs as inputs, doing neural calculations, and producing the V_LS_ as the output. These calculations consist of determining appropriate weights and biases. In the hybrid models, this task is carried out by an optimization algorithm. The organized MLP is decomposed and through an iterative effort, the intended optimization algorithm finds weights and biases for it, reconstructs the MLP, and calculates its training error (MSE). This error represents the objective function of the model. For a certain number of iterations, this process is repeated and the optimization algorithm aims to minimize the objective function by providing a better solution (i.e., a matrix of weights and biases). This process is shown in Fig. 4.

## Results and discussion

### Optimal models

**Figure 5:**
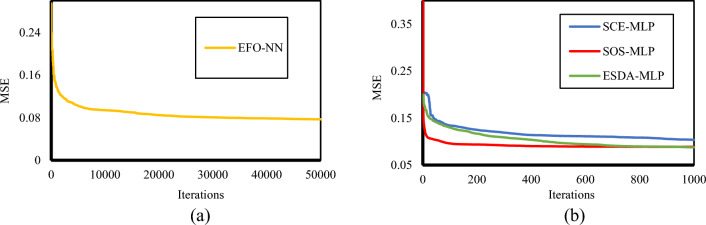
Optimization of (**a**) EFO-MLP and (**b**) SCE-MLP, SOS-MLP, and ESDA-MLP.

In this section, the training results of the EFO-MLP, SCE-MLP, SOS-MLP, and ESDA-MLP are presented. As explained in Section "Optimization algorithms", the training process of hybrid models is an iterative effort. Considering the optimization behavior of each algorithm, a total of 50,000 iterations were deployed for the EFO-MLP, while the other three models could achieve the optimal solution within 1000 iterations. When the calculated objective functions (i.e., MSEs) of all iterations are depicted, the outcome is a curve that illustrates the optimization path. Figure 5 shows the optimization path of the used models.

The selected population sizes for the EFO-MLP, SCE-MLP, SOS-MLP, and ESDA-MLP were 35, 10, 500, and 400, respectively. According to Fig. 5, all models have performed a successful optimization, due to the minimized training errors. Final MSEs corresponding to the EFO-MLP, SCE-MLP, SOS-MLP, and ESDA-MLP were 0.0769, 0.1039, 0.0889, and 0.0876, respectively.

### Creating susceptibility maps

**Figure 6:**
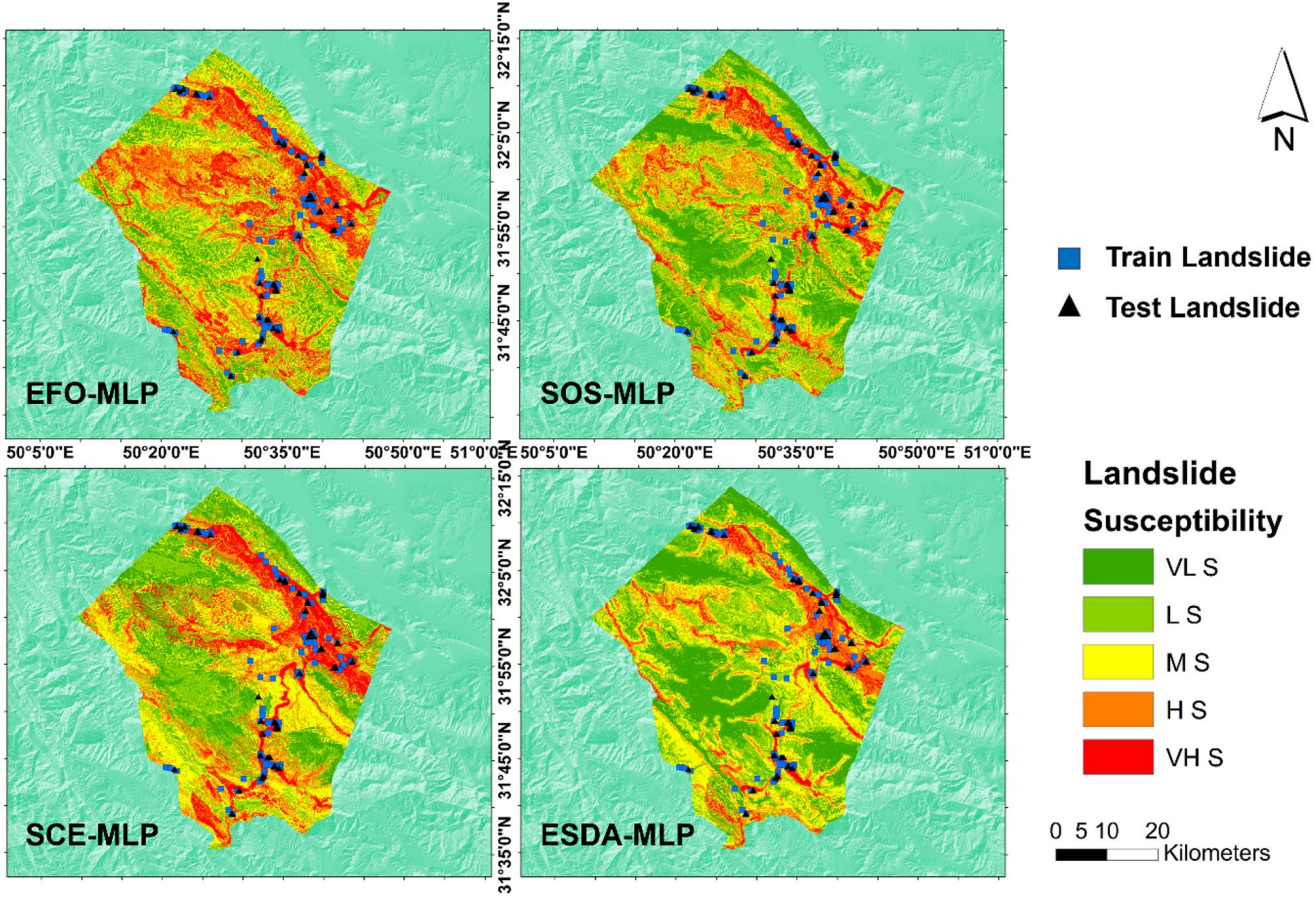
Produced landslide susceptibility maps—created using ArcGIS v10.5^54^. (VL S: very low susceptibility, L S: low susceptibility, M S: moderate susceptibility, H S: high susceptibility, and VH S: very high susceptibility).

The four trained models used training landslide events to attain a desirable level of accuracy with reference to the calculated MSEs. Therefore, these models are ready to extrapolate the learned pattern to the whole study area. The outcome of this process is a landslide susceptibility map that is created by predicting the V_LS_ for all pixels. Therefore, in order to achieve thematic susceptibility maps, a classification method must be applied. In this study, the natural break classification method has been applied which is a popular technique as per many earlier works^72,73^. As the outcome, five classes label the whole area as very low, low, moderate, high, and very high susceptibility to landslide. Figure 6 illustrates the produced maps.

**Figure 7:**
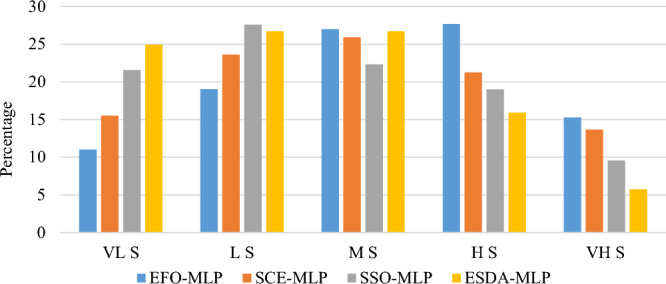
Ratio of the covered susceptibility levels.

As is seen, the generated susceptibility maps have identified VH S and H S for the areas with earlier landslide events. Additionally, in all maps, the Northern-Central parts of the area are labeled as highly susceptible areas. It is highlighted in the map of EFO-MLP map. Also, all models agree on the low susceptibility of the Western-Central parts of the studied area. Figure 7 shows the ratio of the areas labeled by each susceptibility level. Based on this figure, all model agree that majority of the study area is labeled by L S, MS, and H S. Considering the hazardous parts (M S + H S + VH S), the EFO-MLP, SCE-MLP, SOS-MLP, and ESDA-MLP report nearly 70%, 61%, 51%, and 48% of the area, respectively.

### Accuracy evaluation

**Figure 8:**
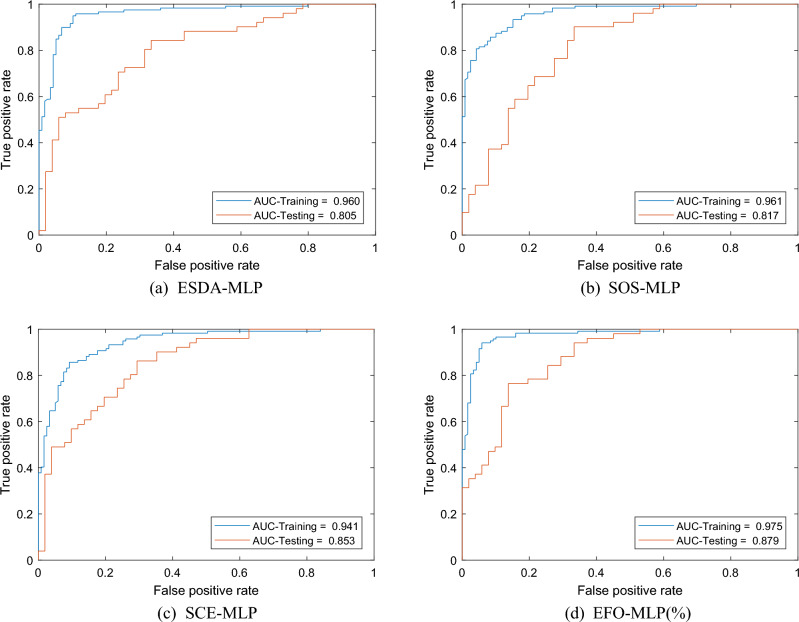
The ROC diagrams of the training and testing data of each model.

Accuracy assessment is a necessary step that must follow landslide susceptibility maps to evaluate the level of accuracy for the training and testing landslides. The results of this section address the reliability of the produced maps. As per Sect. 3.3, five statistical criteria that are considered for this purpose are AUC, specificity, sensitivity, R and MSE. Figure 8 shows the ROC diagrams for each model. As is known, the area beneath each curve gives the AUC values. In a glance, all four models cover a large area indicating excellent accuracy for both training (> 90%) and testing (> 80%) phases. The obtained values will be discussed in the following.

**Figure 9:**
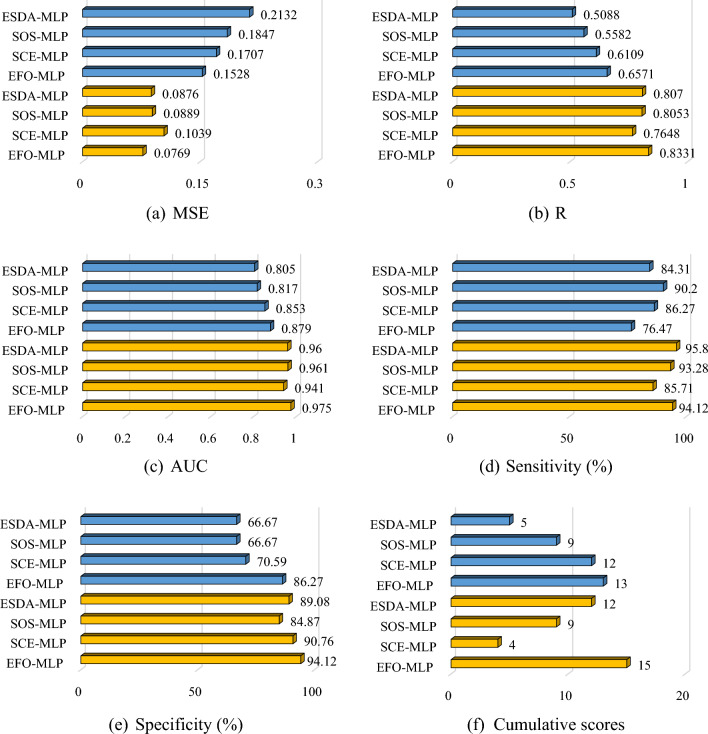
Accuracy criteria results.

Figure 9 shows the bar charts of the calculated AUC, specificity, sensitivity, R and MSE in both training and testing phases. According to Fig. 9, all models have achieved a favorable level of accuracy in both the training and testing phases. For instance, it is seen that the AUC values of all used models are above 0.80. Concerning the training phase, the calculated AUCs were 0.975, 0.941, 0.961, and 0.960 for the EFO-MLP, SCE-MLP, SOS-MLP, and ESDA-MLP, respectively. These values are associated with the specificities of 94.12, 90.76, 84.87, and 89.08, sensitivities of 94.12, 85.71, 93.28, and 95.80, as well as the MSEs of 0.0769, 0.1039, 0.0889, and 0.0876, indicating an excellent training by analyzing the relationship between the LCFs and V_LS_. Moreover, the *R* values of 0.8331, 0.7648, 0.8053, and 0.8070 represent a high agreement between the target and output V_LS_ values. In MLP applications, when the hybrid model receives high-quality training, it means that the weights and biases have been suitably chosen to tune the model. Hence, it can be concluded that the EFO, SCE, SOS, and ESDA are nice options for training an MLP. As for the testing results, the calculated AUCs were 0.879, 0.853, 0.817, and 0.805 for the EFO-MLP, SCE-MLP, SOS-MLP, and ESDA-MLP, respectively. These values are associated with the specificities of 86.27, 70.59, 66.67, and 66.67, sensitivities of 76.47, 86.27, 90.20, and 84.31, as well as the MSEs of 0.1528, 0.1707, 0.1847, and 0.2132, indicating a reliable prediction of V_LS_ for the whole study area. Moreover, the *R* values of 0.6571, 0.6109, 0.5582, and 0.5088 represent a high agreement between the target and output V_LS_ values. From the computational point of view, it means that the chosen weights and biases have created a generalizable model in all cases. Therefore, the created susceptibility maps are reliable and they can address critical regions.

### Comparison

Having the values of five accuracy criteria (i.e., MSE, R, AUC, sensitivity, and specificity), it is possible to compare the performance of the applied models. Figure 9a–e compare the obtained accuracy criteria in the form of bar charts, while Fig. 9f shows the cumulative scores of each model. For calculating these scores, each accuracy criterion is assessed separately so that a score between 1 and 4 is assigned to each model (the higher the accuracy, the higher the score) and the scores of all accuracy criteria are summed for each model to determine its cumulative score. Hence, a model with a higher cumulative score has shown a better overall performance. For instance, in the training phase, the EFO-MLP obtains a value of 15 because it has the best accuracy in terms of the MSE, R, AUC, and specificity, while it is second-best in terms of sensitivity (4 + 4 + 4 + 4 + 3 = 15).

In the training phase, the ESDA-MLP is the second-best model with a cumulative score of 12, followed by the SOS-MLP and SCE-MLP with respective cumulative scores of 9 and 4. As for the testing phase, the EFO-MLP again stands first with a cumulative score of 13. However, this time the SCE-MLP gains the second position with a cumulative score of 12. Considering the cumulative scores of 9 and 5, the SOS-MLP and ESDA-MLP are ranked as the third and fourth models, respectively.

Another factor to be considered for comparison is the optimization time. This process is an iterative effort that requires implementing a large number of iterations. For optimizing the MLP model in this study, the EFO algorithm took around 1161 s for 50,000 iterations, while the SCE, SOS, and ESDA took about 715, 91,293, and 2745 s, respectively, for 1000 iterations.

Overall, due to the highest accuracy in both the training and testing phases, as well as owing to the fast optimization capability, the EFO-MLP can be selected as the outstanding model of this study. However, it should be noted that the SCE-MLP attained very good testing results as well, along with having the fastest optimization.

### V_LS_ formula

According to the explanations in Section "MLP neural network" and "Optimization algorithms", the internal parameters of the MLP model (i.e., weights and biases) were adjusted using optimization algorithms (EFO, SCE, SOS, and ESDA). On the other hand, it was demonstrated that the EFO-MLP was the superior model. Hence, it would be advantageous if we could develop a mathematical equation upon this model. Since the calucaltions were carried out using the MATLAB environment, two commands of “getwb()” and “separatewb()” were used to extract the weights and biases. They are then arranged to create a two-part mathematical equation. First, Eq. 6 and Table 2 apply the weights and biases (i.e., *W*_*1,I*_*, W*_*2,i*_* , …, b*_*i*_) to the LCFs and achieve *N*_1_*, N*_2_*, N*_3_*, N*_4_*,* and* N*_5_. Next, these five values are used in Eq. 7 to calulate V_LS_.

6 $$N_{i} = \, Tansig(W_{1,i} \times Elevation + W_{2,i} \times \, D. \, \;to\;Faults + W_{3,i} \times \, D.\;to\;Rivers + W_{4,i} \times D.\;to\;Roads + W_{5,i} \times Land\;Use + W_{6,i} \times Soil\;Type + W_{7,i} \times Lithology + W_{8,i} \times TWI + W_{9,i} \times \, SPI + W_{10,i} \times Plan\;Curvature + W_{11,i} \times Profile\;Curvature + W_{12,i} \times Slope\;Degree + W_{13,i} \times Slope\;Aspect + W_{14,i} \times NDVI + b_{i} )$$

7 $${\text{V}}_{{{\text{LS}}}} = \, Purelin( - 0.998763 \, \times N_{1} - \, 0.044217 \, \times N_{2} - \, 0.944105 \, \times N_{3} + \, 0.132491 \, \times N_{4} + \, 0.060674 \, \times N_{5} - \, 0.897453)$$

where *Tansig(x)* = $\frac{2}{{1 + e^{ - 2x} }} - 1$ and *Purelin(x)* = *x* are the activation functions.

**Table 2:** EFO-MLP’s internal parameters (for Eq. 6).

i	1	2	3	4	5
W_1,i_	9.624480	− 2.430336	− 2.912813	0.754190	0.713455
W_2,i_	− 1.222415	1.755621	0.908715	− 2.338748	− 0.639343
W_3,i_	0.603414	2.394439	0.223872	3.055910	− 1.011313
W_4,i_	3.606757	4.021633	1.016440	− 0.767411	− 1.009544
W_5,i_	0.239853	0.904416	2.040009	3.227112	− 2.390894
W_6,i_	− 1.272790	2.719250	0.196339	2.587112	0.776340
W_7,i_	− 3.175586	1.142060	1.714491	0.658868	2.157040
W_8,i_	0.071876	− 0.176647	− 0.616245	− 1.818853	1.655055
W_9,i_	− 0.081560	− 1.017455	1.076561	0.522834	− 0.221117
W_10,i_	− 0.041508	2.360388	− 0.066176	− 0.020062	− 1.995437
W_11,i_	− 0.296002	− 1.870286	− 0.175865	− 1.211587	3.490253
W_12,i_	0.673016	1.544421	0.599220	− 1.300808	1.286953
W_13,i_	− 0.029455	0.812452	− 0.770832	− 0.918721	1.138201
W_14,i_	1.751352	1.651923	− 1.835972	4.982407	1.009059
b_i_	− 6.141255	0.643019	− 0.872081	1.240851	− 1.379554

**Figure 10:**
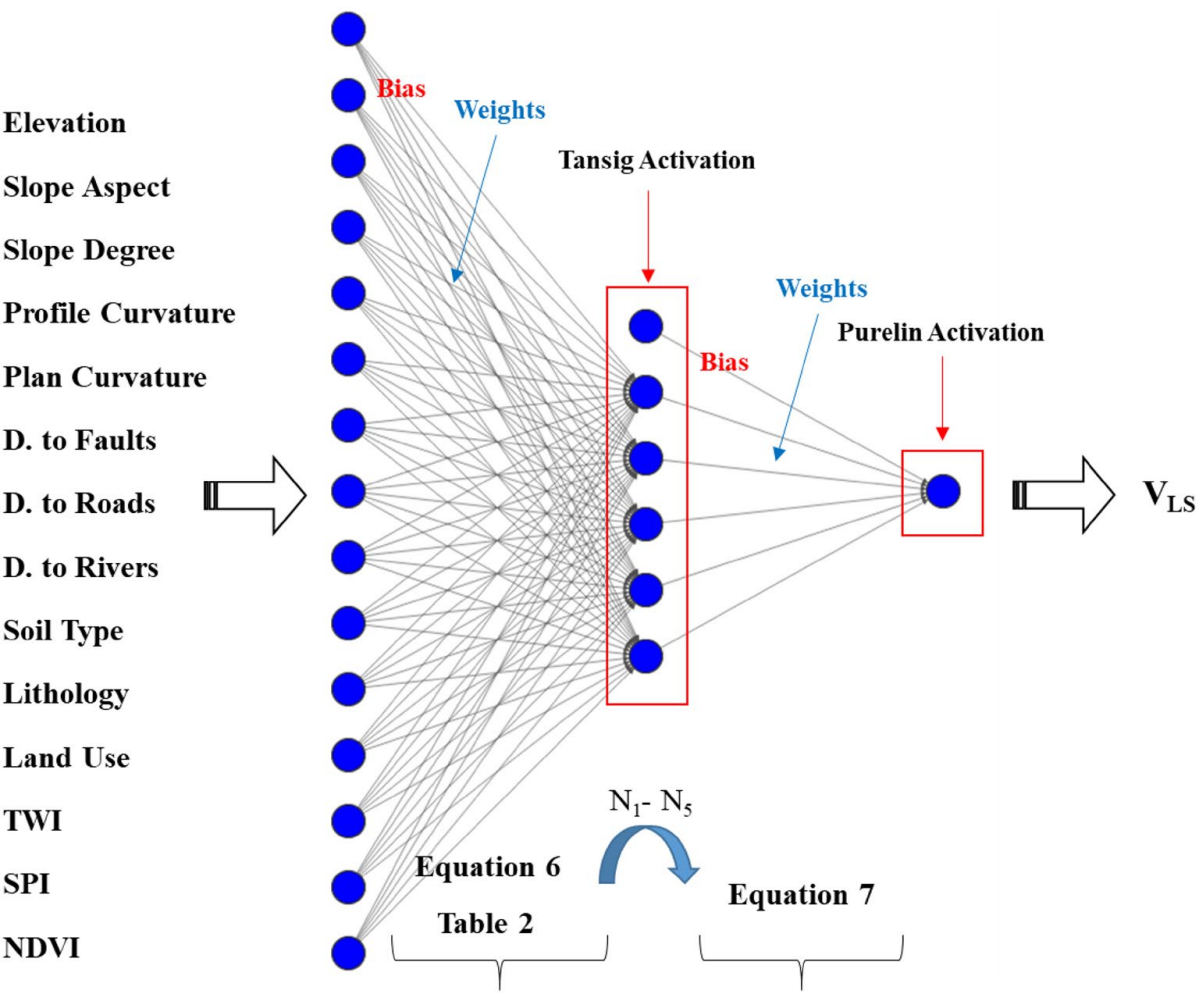
V_LS_ Formula composition based on MLP.

Figure 10 shows how the formula corresponds to the previous explanations. The EFO-MLP first receives the LCFs as inputs; *N*_1_*, N*_2_*, N*_3_*, N*_4_*,* and* N*_5_ are produced in the middle layer and transmitted to the output layer to play the role of secondary inputs in this layer.

### Further discussion

From Fig. 1 and produced susceptibility maps, it can be inferred that many human residences (i.e., villages) fall within areas with dangerous level of landslide susceptivility. It calls for applying risk analysis by considering hazard, exposure, and vulnerability and establishing proper protective measures. For instance, landslide warning systems (i.e., mass movement sensors) and protective shields must be installed within prone slopes along the roads. Additionally, Fig. 2 revealed the more important role of Elevation, Lithology, and Land Use among the considered LCFs. Hence, relevant authorities are expected to pay higher attention to the areas with specific characteristics of these three factors.

From computational point of view, this study achieved improvements compared to previous research. For instance, notwithstanding a simpler MLP model in the present research, the EFO algrithm achieved a higher accuracy compared to chimp optimization algorithm (ChOA) and crow search algorithm (CSA) employed by Mehrabi and Moayedi^49^. The AUCs of the ChOA and CSA were 0.851 and 0.855 which are smaller than 0.879 which is recorded for the EFO. Moreover, the SCE in this study could accomplish the optimization task in a much lower time (715 s vs. 1064 s and 13,320 s—with almost similar computer systems).

Another advantage of this study is concerned with the V_LS_ mathematical formula and executed importance assessment. The introduced formula can be directly used to predict the V_LS_ without the need for performing heavy and complicated computer programming. Moreover, the carried-out RFIA (Fig. 2) can shed lights on understanding the role of LCFs more clearly. The results of that part can be used in future efforts to guide the authors for feature analysis and simplifying burdensome computations due to the large number of involved LCFs.

Another viable idea for continuing landslide risk assessment in this area is taking dynamic factors into account. For instance, rainfall is one of the factors that plays a significant role in landslide occurrence. Provided with a reliable prediction of rainfall in the future, one may attain a dynamic landslide susceptibility prediction model.

## Conclusions

The significance of using landslide susceptibility maps for risk management of prone areas is evident. This study, therefore, was dedicated to developing new integrative models based on artificial neural network for the prediction of landslide susceptibility in Ardal County, West of Iran. A comprehensive database was used to consider the effect of many landslide conditioning factor. Four models were compared in terms of accuracy indicators and time-efficiency and according to the results, the EFO-MLP turned out to be the most accurate and the second-fastest predictive model. The maps developed in this study could nicely label the landslide-prone areas with high accuracy which may be regarded for designing protective measures. An importance assessment revealed the role of each LCF which can be of great help for the mentioned task. Moreover, the suggested model was also simplified into a mathematical format for an easier calculation of landslide susceptibility without sophisticated tools. For future studies, it is suggested to expand the findings of this study to a comprehensive hazard assessment with respect to the exposure and vulnerability of the people and assets within the studied area.

## Data Availability

All data generated or analysed during this study are can be avialble upon reasonable request from the author Shangshang Xu.
